# Annurca Apple Polyphenols Ignite Keratin Production in Hair Follicles by Inhibiting the Pentose Phosphate Pathway and Amino Acid Oxidation

**DOI:** 10.3390/nu10101406

**Published:** 2018-10-02

**Authors:** Nadia Badolati, Eduardo Sommella, Gennaro Riccio, Emanuela Salviati, Dimitri Heintz, Sara Bottone, Emery Di Cicco, Monica Dentice, Giancarlo Tenore, Pietro Campiglia, Mariano Stornaiuolo, Ettore Novellino

**Affiliations:** 1 Department of Pharmacy, University of Naples Federico II. Via Montesano 49, 80149 Naples, Italy; badolatin@gmail.com (N.B.); genriccio@gmail.com (G.R.); sara.bottone@unina.it (S.B.); giancarlo.tenore@unina.it (G.T.); 2 Department of Pharmacy, School of Pharmacy, University of Salerno, Via Giovanni Paolo II 132, I-84084 Fisciano, Italy; esommella@unisa.it (E.S.); esalviati@unisa.it (E.S.); pcampiglia@unisa.it (P.C.); 3 PhD Program in Drug Discovery and Development, University of Salerno, Via Giovanni Paolo II 132, I-84084 Fisciano, Italy; 4 Plant Imaging and Mass Spectrometry, Institut de Biologie Moleculaire des Plantes, CNRS, Universite de Strasbourg, 67000 Strasbourg, France; dimitri.heintz@ibmp-cnrs.unistra.fr; 5 Department of Clinical Medicine and Surgery, University of Naples Federico II, Via Pansini 5, 80149 Naples, Italy; emery2304@gmail.com (E.D.C.); monica.dentice@unina.it (M.D.)

**Keywords:** nutraceuticals, apple polyphenols, Procyanidin B2, anti-oxidants, hair growth

## Abstract

Patterned hair loss (PHL) affects around 50% of the adult population worldwide. The negative impact that this condition exerts on people’s life quality has boosted the appearance of over-the-counter products endowed with hair-promoting activity. Nutraceuticals enriched in polyphenols have been recently shown to promote hair growth and counteract PHL. *Malus pumila Miller* cv. Annurca is an apple native to Southern Italy presenting one of the highest contents of Procyanidin B2. We have recently shown that oral consumption of Annurca polyphenolic extracts (AAE) stimulates hair growth, hair number, hair weight and keratin content in healthy human subjects. Despite its activity, the analysis of the molecular mechanism behind its hair promoting effect is still partially unclear. In this work we performed an unprecedented metabolite analysis of hair follicles (HFs) in mice topically treated with AAE. The metabolomic profile, based on a high-resolution mass spectrometry approach, revealed that AAE re-programs murine HF metabolism. AAE acts by inhibiting several NADPH dependent reactions. Glutaminolysis, pentose phosphate pathway, glutathione, citrulline and nucleotide synthesis are all halted in vivo by the treatment of HFs with AAE. On the contrary, mitochondrial respiration, β-oxidation and keratin production are stimulated by the treatment with AAE. The metabolic shift induced by AAE spares amino acids from being oxidized, ultimately keeping them available for keratin biosynthesis.

## 1. Introduction

During its life, a human healthy hair follicle (HF) undergoes cyclical rounds of growth, active fiber production (anagen), degeneration (catagen) and rest (telogen) [1]. Anagen, the phase when hair shaft extension occurs, can last years in humans and is fueled by hair matrix cells (HMs) located in the hair bulb (HB). These highly proliferating cells are stimulated by the dermal papillas (DP) to differentiate into different lineages of cell including the outer (ORS) and inner (IRS) root sheaths, all differently involved in the production, deposition, protection and hardening of keratins [1,2,3,4].

Patterned hair loss (PHL) affects around 50% of the adult population worldwide. Independent of age and gender, this condition exerts a profound negative impact on some people’s quality of life [4] and, especially when occurring at an early age, it is frequently associated to depression [5,6]. During PHL, HFs undergo a premature miniaturization and shorten their permanence in anagen. The etiology of PHL is complex and involves a combination of genetic, psychological, and environmental factors [7,8]. Only for Androgenic Alopecia, one of the most common cause of PHL, pathogenesis has been partially elucidated and proven to involve an altered metabolism of testosterone [6]. The increased activity of the type II isoform of the 5-α reductase enzyme (5AR) and the local level of its product dihydrotestosterone, results in shortening of anagen phase and progressive miniaturization of the HFs [9].

Finasteride (FIN), the only United States Federal Drug administration (FDA) approved oral agent for PHL, is a specific inhibitor of 5AR type II isoform. Minoxidil (MIN), the other FDA approved topical agent acts as potassium channel opener, apparently increasing follicular vascularity of the hair DP, prolonging anagen and shortening telogen [10]. Considering the occurrence and the complex etiology of PHL, it is widely accepted that new therapies have to be identified [11]. Moreover, FIN and MIN activity reaches a plateau within two years of usage and both produce adverse effects on patients [12,13].

In the past decade, an increasing number of reports have provided support for nutritional and antioxidant therapies as effective and safe treatment options for hair loss. Dietary supplements like those enriched in plant extracts [14,15], amino acids [16,17], vitamins [18] are popular over-the-counter products and have indeed been shown to increase anagen rate in PHL patients. Nutraceuticals enriched in Procyanidin B2, a dimeric procyanidin, have been recently shown to promote hair growth and induce anagen in humans [19]. *Malus pumila* cv. Annurca, an apple native to Southern Italy, presents one of the highest contents of Procyanidin B2 [20]. Recently we have published the results of a clinical trial describing the effect of Annurca Apple polyphenolic extract of (AAE) on hair growth in healthy subjects. A significant result in terms of increased hair growth, hair number, hair weight and keratin content could be measured already after 2 months of oral treatment [21].

To date, despite the increased usage of nutraceuticals for hair growth, studies concerning their mechanism of action are far from being complete. Nutraceuticals are supposed to generally act either as antioxidants or indirectly by increasing vascularization, blood flow and nutrient supply to the HFs, ultimately affecting their homeostasis and metabolism [22,23,24,25]. As for many other polyphenols, the molecular mechanism underpinning AAE activity on HFs is still obscure. Moreover, the hundreds of polyphenols contained in AAE [26] influence each other complicating the analysis of the intracellular pathway influenced by the extract. In vitro, AAE stimulates proliferation of keratinocytes and induces production of keratins [21]; in murine epithelial cells, pure Procyanidin B2 has been shown to inhibit Protein Kinase C (PKC) isozymes [27] and modulate transforming growth factor Transforming Growth Factor β [19,27,28,29,30,31].

To give new insights into the growth promotional effects exerted by AAE on HFs, we here investigated the metabolomic profile of murine HFs treated with AAE. C57BL/6 mice received a topical treatment with a cosmetic foam containing AAE. Upon 4 weeks of treatment, murine HFs were extracted from the skin and their metabolome were analyzed by Direct Infusion Fourier Transform-ion cyclotron resonance mass spectrometry (DI-FT-ICR-MS). This technique is characterized by unmatched ultra-high mass accuracy and resolution, that make it highly suitable in metabolite profiling [32]. Metabolomic approaches are extremely useful tools for probing any change in metabolism accompanying drug treatments and provide invaluable insights in the mechanism of action of complex mixtures and phytocomplexes [33]. Measuring the specific effect that AAE exerted on the metabolome of HF cells we can here show that the polyphenolic extract is able to stimulate keratin production in HFs by shifting HF metabolism from aerobic glycolysis and glutaminolysis into oxidative mitochondrial respiration.

## 2. Materials and Methods

### 2.1. Reagents

Chemicals and reagents used for metabolite extraction were all HPLC grade. Water was treated in a Milli-Q water purification system (Millipore, Bedford, MA, USA) before use. The standards used for the identification of intracellular HF metabolites were from Sigma Chemical Co. (St. Louis, MO, USA). Haematoxylin and Eosin were provided by Bio-Optica (Milan, Italy). MitoTracker Red CMXRos (M7512, Invitrogen, Carlsbad, CA, USA) used for ex vivo staining of mitochondria in skin biopsies was reconstituted in DMSO and 1 mM stock aliquots were stored at −20 °C before use.

### 2.2. Nutraceuticals 

Composition of AAE cosmetic foam: AnnurtriComplex (industrial procyanidinic extract of Annurca apple PGI (Protected Geographical Indication) (*Malus pumila Miller* cv. Annurca) produced by MB-Med (Turin, Italy)) 6% (*w*/*v*), water, glycerin, decylglucoside, polysorbate, maltodextrin, potassium sorbate, sodium benzoate, silica. The Placebo foam was formulated identically but did not contain AAE.

### 2.3. Animals

Wild-type C57BL/6 mice (7 weeks old, postnatal day 49) were used in all experiments to test the effect of cosmetic foam containing AAE. All animals received human care and were maintained in separate cages at 22–24 °C and fed a general rodent diet. Differently from other published protocols, here animals were left unshaved and received a topical treatment with 2 cm^3^ of the indicated cosmetic foam (this would correspond to 2–4 mL of foam for an adult human scalp) for 4 weeks, twice a week. Only male animals were used in this study. All animal experiments were performed in compliance with ethical guidelines and approved by the University of Naples Federico II.

### 2.4. Histology 

After 4 weeks of treatments with AAE, mice were sacrificed and their dorsal skin immediately excised and immersed in Phosphate Buffer Saline (PBS). 1 cm^2^ of skin biopsies were fixed overnight in 4% formalin, washed in PBS, dissected and embedded in paraffin. 5–10 μm sections were deparaffinised and stained with haematoxylin and eosin using standard procedures. For ex-vivo staining of mitochondria, mice skin biopsies, immediately after excision, were rinsed in PBS and located in six multi-well plates. The staining solution was prepared by diluting MitoTracker CMX-Ros in Dulbecco Modified Eagle Medium (DMEM) (Invitrogen, Carlsbad, CA, USA) (without fetal bovine serum) to yield a final concentration of 200 nM [34]. Tissues were incubated for 40 min in a Cell Culture incubator at 37 °C, supplemented with 5% CO_2_. At the end of the incubation, tissues were rinsed three times in DMEM, fixed in 4% formaldehyde (diluted in PBS pH 7.4), embedded in Paraffin or in OCT and sectioned with a Leica 3200 Cryostat (Ramsey, MN, USA). 10–20 μm sections were washed in 96%, 90% and finally in 80% EtOH solution (5 min wash) to be then covered with a glycerol/PBS solution 1:1 (to avoid drying) and visualized under a fluorescent microscope using the following antibodies and as already described [35]. When indicated sections were processed for immunofluorescence using the following antibodies: anti-Keratin14 (cod. D14IF01918, Covance, Princeton, NJ, USA), anti-Keratine6 (cod. PRB-169P, Covance, Princeton, NJ, USA). Scanning Electron Microscopy (SEM) and Energy Dispersive X-Ray (EDX) analysis of hair shafts were performed with a bench top Phenom XL (Alfatest, Milan, Italy) following manufacturer instructions and as already described [36].

### 2.5. Metabolite and RNA Extraction from Murine Tissues

Immediately after excision, tissues were rinsed and kept in PBS. Hair shafts were plucked out with a sterile tweezers and immediately covered with a solution of PBS at R.T.(Room Temperature) To allow detachment of HF cells, plunked HFs were incubated for 15 min in PBS supplemented with 5 mM EDTA. Hair shafts were removed with a cell strainer and HF cells were centrifuged for 5 min at 500 rpm. The cell pellets were washed twice in PBS and split in two aliquots. The first pool of cells was solubilized in 1 mL of Trizol (Invitrogen, Carlsbad, CA, USA) and stored at −80 °C for qPCR analysis [35]. The second pool was homogenized in 1 mL of pre-chilled methanol/water 1:1 solution containing 10 nmoL of internal standard and finally centrifuged at 10,000× *g* for 10 min at 4 °C [37]. The resulting supernatants were collected and transferred into new Eppendorf tubes and stored at −80 °C.

### 2.6. Mass Spectrometry-Based Metabolomic, Statistics and Analysis

Analyses were performed in direct infusion following a previous protocol [32,38] employing a Hamilton syringe (250 μL) at a flow rate of 2 μL/min. Data were acquired on a SolariX XR 7T (Bruker Daltonics, Bremen, Germany). The instrument was tuned with a standard solution of sodium trifluoracetate. Mass Spectra were recorded in broadband mode in the range 100–1500 *m*/*z*, with an ion accumulation of 20 ms, with 32 scans using 2 million data points (2 M). Nebulizing (N_2_) and drying gases (air) were set at 1 and 4 mL/min, respectively, with a drying temperature of 200 °C. Both positive and negative ESI (Electro Spray Ionization) ionization were employed. Five replicates of each injection were carried out. The instrument was controlled by Bruker FTMS Control, MS spectra were elaborated with Compass Data Analysis version 4.2 (Bruker, New York, NY, USA), identification of compounds based on accurate MS measurements was performed by Compound Crawler version 3.0 and Metaboscape 3.0 (Bruker, New York, NY, USA). Metabolites signals were normalized using internal standards. Comparisons and differences were analyzed for statistical significance by two-way Anova test and Bonferroni post-tests analysis. All graphs, bars or lines indicate mean and error bars indicate standard error of the mean (SEM). Furthermore, Partial Least Square Discriminant Analysis (PLS-DA) was used as classification model. All graphs, bars or lines indicate mean and error bars indicate standard error of the mean (SEM). Statistical analysis was performed using Statistica software (StatSoft, Tulsa, OK, USA) and Minitab (Minitab Inc., State College, PA, USA).

## 3. Results

### 3.1. Topical Treatment with AAE Accellerates Exit of Murine HF from Telogen and Increases Keratin Content in Hair Shafts

**Figure 1:**
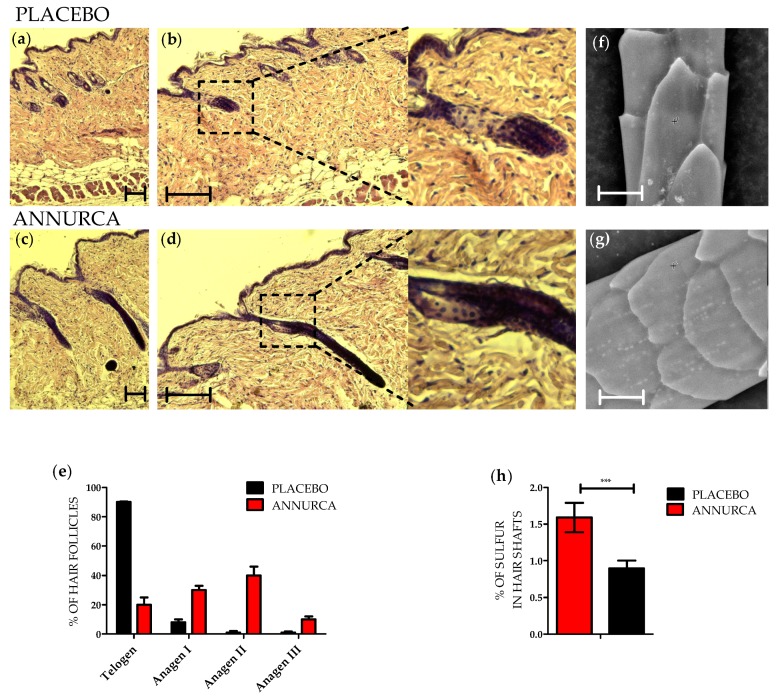
AAE induces early exit from telogen and increases keratin content of hair shafts in murine HFs. C57BL/6 mice were treated topically with a foam supplemented either with AAE or with a placebo. Foams were applied on the dorsal skin of 7-week-old mice. After 4 weeks of treatment, mice were sacrificed and HFs classified following morphological criteria. Haematoxylin-eosin staining of HFs of mice treated with placebo (**a**–**b**) showing HFs mostly in Telogen/AnagenI phase. HFs of mice treated with AAE appeared in a later stage of Anagen, mostly Anagen II (**c**–**e**). (**f**–**h**) SEM (SEM-EDX) analysis of hairs extracted from mice treated with placebo (**f**) or with AAE (**g**). SEM quantitative analysis indicates (**h**) an increase in Sulfur concentration (cystine, methionine, cysteine and cysteic acid all abundant amino acids of hair keratins) in hairs of mice treated with AAE. Values in € and (**h**) are reported as mean ± SEM (*n* = 8, *** *p* < 0.001). Scale bars in a-d correspond to 200 μm. Scale bars in (**f**) and (**g**) correspond to 10 μm.

C57BL/6 mice were treated topically with a foam supplemented either with AAE (see methods for details) or with a placebo. Foams were applied on the dorsal skin of 7-week-old mice. At the beginning of the treatment, all the mice presented pink skins, indicating their HFs being in telogen [39]. After 4 weeks of treatment, mice (11-week-old) were sacrificed and their dorsal skin excised. Skin biopsies were embedded in paraffin and prepared for histology. HFs were classified following morphological criteria into the different stages of the hair cycle [40,41]. As expected, HFs of mice treated with placebo were mostly in Telogen/Anagen I phase (Figure 1a,b). HFs appeared mainly located in the dermis with only few DPs touching the subcutis border. Moreover, they appeared fully surrounded by inter-follicular dermal fibroblasts and presented ORS but not IRS. Differently, HFs of mice treated with AAE appeared increased in length and were located closer to the subcutis (Figure 1c,d). Quantitation indicated HFs of mice treated with AAE being in Anagen, most of them in Anagen II and few in Anagen III (Figure 1e).

To confirm HFs of mice treated with AAE being in a hair shaft production phase of the hair cycle, keratin content and microstructure of the hair shaft sections closest to the HFs were evaluated by SEM-EDX [36]. The percentage of Sulfur in the hair shaft was measured and correlated to the content of keratins (cystine, methionine, cysteine and cysteic acid are abundant amino acids of the hair). Compared to placebo, AAE-treated hairs showed an increased content of Sulfur and thus of keratins (Figure 1f–h). Since keratins are only produced by anagen HFs, our SEM data confirm histochemical analysis indicating that AAE topical treatment accelerated exit of murine HFs from telogen phase.

### 3.2. Topical Treatment with AAE Alters the Intracellular Levels of HF Key Metabolites

HF cells were plucked out of mice treated topically with AAE and their mRNAs analyzed by quantitative Real-time qPCR. As shown in Figure S1, AAE reduces the expression of CD34, a marker of quiescent, infrequently dividing, murine bulge stem cells [42]. Differently, AAE has less effect on the transcription of Keratin 15, another marker of bulge stem cells and of Lgr5 a Wnt target gene. Interesting GLI-1, that is barely expressed in HF cells treated with Placebo is strongly induced by AAE [43]. GLI-1, is a marker of hair bulge and hair germ. Stem cells expressing GLI-1 have been shown to give rise to differentiated cells in all layers of the HF. The increased GLI-1 expression confirmed AAE inducing exit of HFs from telogen, since GLI-1 positive cells have been reported to be the first triggered at the onset of anagen [43].

The metabolic content of HF cells plucked out of mice treated topically with AAE were analyzed by DI-FT-ICR mass spectrometry. qPCR analysis confirmed that most of the HFs cell lineages are represented in our samples. It must be taken in account, however, that we cannot ascertain their relative contribution to the metabolic analysis here presented.

Complex profiles were obtained in both positive and negative ionization. The high mass accuracy (average: 0.166 ppm, Table S1), isotopic distribution and comparison with available standards, ensured confident identification. As can be appreciated from Figure S2, showing the scores plot of PLS-DA in both positive and negative ionization, a distinct separation between AAE-treated and Placebo HF metabolomes can be observed. By screening for intracellular metabolites with similar alteration tendency in all the AAE-treated mice, glutaminolysis, pentose phosphate pathway (PPP), amino acid oxidation, mitochondrial β-oxidation and arginine metabolism became our focus.

**Figure 2:**
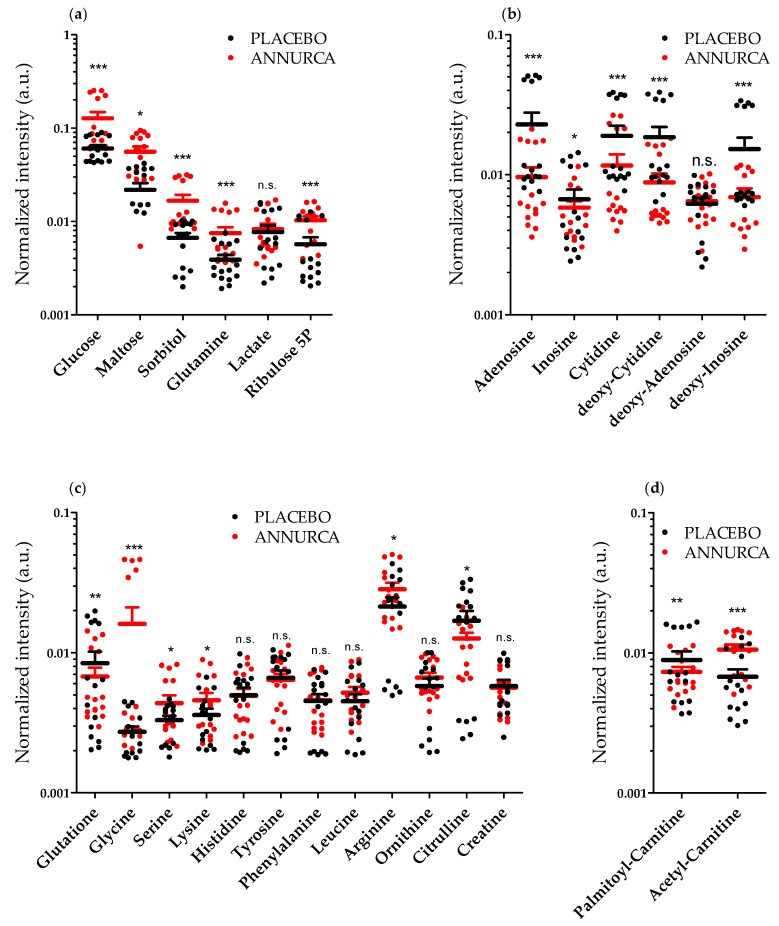
AAE diverts HF metabolic pathways from PPP and amino acid oxidation. Metabolomic analysis (Carbohydrates (**a**); nucleotides (b); amino acids (**c**); carnitine derivatives (**d**)) of HF cells extracted from C57BL/6 mice treated topically for 4 weeks with a foam containing AAE (red dots, ANNURCA) or a Placebo (black dots PLACEBO). Each point represents the normalized intensity of the metabolite (*n* = 15 measurements, mean ± SEM. Two way ANOVA and Bonferroni post-test analysis were performed; * = *p* < 0.05; ** = *p* < 0.01; *** = *p* < 0.001; n.s.: non statically different).

In mammals, HF metabolism is mainly set on aerobic glycolysis and glutaminolysis [44]. Despite the presence of oxygen and of functional mitochondria, HF cells obtain energy mostly by converting glucose into lactate [44]. This product of cellular fermentation is secreted by HF cells and reaches the liver (via the blood circulation) to be recycled back to glucose via the Cory cycle. We thus started our metabolic profiling looking for statistically significant changes in the levels lactate, glucose and related sugars. We measured a significant elevation in the intracellular levels of both glucose (2.4 ± 0.1-fold, mean ± SEM, *p* < 0.001) as well as of the glycogen synthesis/degradation products maltose (2.2 ± 0.2-fold, *p* = 0.032) and sorbitol (3.1 ± 0.1-fold, *p* < 0.001) (Figure 2a). On the contrary, lactate levels were not significantly altered by AAE (1.1 ± 0.2-fold, *p* > 0.05) (Figure 2a).

This elevation in the intracellular levels of the glucose, maltose and sorbitol is compatible with both a decrease and an increase in the rate of Glycogenolysis. However, since lactic acid, a product of aerobic glycolysis, is not significantly altered by AAE, it is reasonable to suppose an inhibition rather than of an induction of Glycogenolysis.

A considerable percentage of glucose and glutamine are usually oxidized in the HFs via the PPP [44]. Similarly, to glucose, we measured a significant elevation of glutamine (2.4 ± 0.1-fold, *p* < 0.001) in HF cells treated with AAE (Figure 2a).

We could as well measure an increase in the intracellular levels of the PPP intermediate ribulose 5-phosphate (ribulose 5P)/xylulose 5-phosphate (isobaric compounds, 3.2 ± 0.2-fold, *p* < 0.001) suggestive of a reduced oxidation of glucose and glutamine via the PPP.

Matrix cells of the HFs have one of the fastest rates of cell division in the mammalian body. PPP supports nucleotide biosynthesis in order to maintain their concentration sufficiently high to allow DNA and RNA synthesis. Considering the increase in ribulose 5P, we were thus expecting a decrease in the levels of nucleotides and deoxy-nucleotides. We indeed measured (Figure 2b) a decrease in the intracellular levels of adenosine (0.40 ± 0.09-fold, *p* < 0.001), cytidine (0.6 ± 0.1-fold, mean ± SEM, *p* value < 0.001), deoxy-cytidine (0.5 ± 0.1-fold, *p* < 0.001) and deoxy-inosine (0.45 ± 0.12-fold, *p* value < 0.001). On the contrary, inosine (0.9 ± 0.1-fold, *p* < 0.05) and deoxy-adenosine (1.0 ± 0.1-fold, *p* > 0.05) were not significantly altered by AAE (Figure 2b).

Similarly, to glutamine, also the intracellular level of glycine appeared increased upon treatment with the polyphenolic extract (6.2 ± 0.2-fold, *p* < 0.001) (Figure 2c). Both glutamine and glycine [45] inputs are rate limiting steps for the synthesis of the tripeptide (Glu-Cys-Gly) glutathione (GSH), the main intracellular scavenger of Reactive Oxigen Species (ROS). Intracelluar levels of GSH underwent a small but statically significant decrease (0.9 ± 0.1-fold, *p* < 0.005) in HFs treated with Annurca Polyphenols (Figure 2c), further suggesting a reduced conversion of the two amino acids in GSH.

The (i) significant elevation of glutamine and (ii) glycine, (iii) the increase in the intracellular level of the PPP intermediate ribulose 5P together with (iv) the reduction of the intracellular levels of nucleotides and deoxy-nucleotides, all together suggest that AAE causes a reduction in the utilization of glucose and glutamine for PPP, a metabolic pathway that in HFs, supports nucleotide biosynthesis. The reduced intracellular level of GSH confirmed that the catabolism of glutamine is halted in AAE-treated HFs.

The effect exerted by AAE on the intracellular levels of glutamine and glycine reflects that observed for other amino acids (Figure 2c). We measured an increase in the intracellular levels of arginine (1.3 ± 0.2-fold, *p* < 0.05), serine (1.3 ± 0.2-fold, *p* < 0.05), and lysine (1.3 ± 0.2-fold, *p* < 0.05). Differently, the intracellular level of histidine (1.0 ± 0.1-fold, *p* > 0.05), tyrosine (1.0 ± 0.1-fold, *p* > 0.05), leucine (0.9 ± 0.1-fold, *p* > 0.05) and phenylalanine (1.0 ± 0.1-fold, *p* > 0.05) were not statistically altered by AAE. Interesting, glutamine and glycine are important component of hair keratins. The intracellular increase in arginine correlates with a reduction in intracellular level of citrulline (0.7 ± 0.2-fold, *p* < 0.05), but not of the other two arginine derivatives ornithine (1.1 ± 0.2-fold, *p* > 0.05) and creatine (1.0 ± 0.1-fold, *p* > 0.05), whose levels did not change significantly (Figure 2c).

Overall, considering the results of our SEM data and the metabolite profiles we can suggest that AAE diverts the intracellular metabolism of HFs from being mainly set on PPP and amino acid oxidation, leaving a pool of selected amino acids ready to be used for keratin biosynthesis. Considering that glycolysis and glutaminolysis are the main source of energy for hair bulb cells we went looking for metabolites that could suggest a new source of energy compensating for the absence of glucose and glutamine metabolism. Mitochondrial β-oxidation represents an alternative source of energy for HF cells [46,47,48]. In AAE-treated HFs, we measured a significant decrease in the intracellular levels of the β-oxidation substrate palmitoyl carnitine (0.8 ± 0.1-fold, *p* < 0.05) (Figure 2d) suggestive of an increase in mitochondrial β-oxidation activity [49]. Similarly, we measured an increase in acetyl-carnitine (Figure 2d) a metabolite enriched in cells with highly active mitochondria (1.6 ± 0.2-fold, *p* < 0.001) [49].

**Figure 3:**
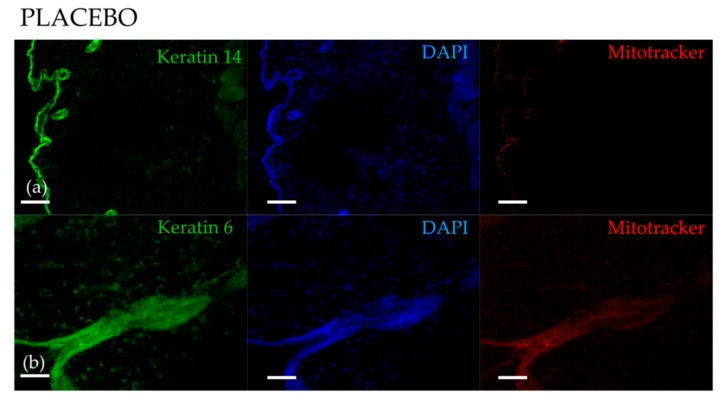

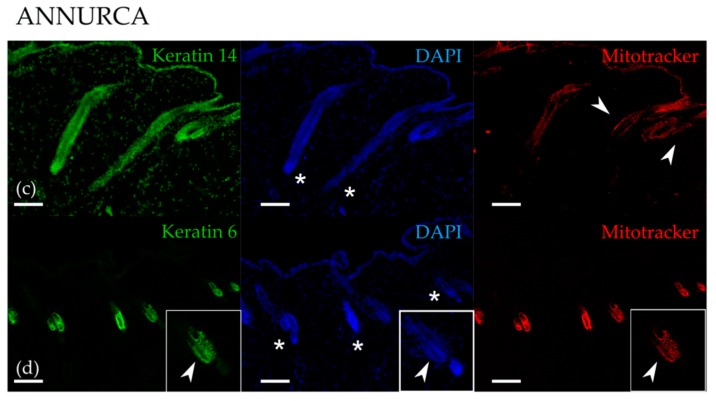
AAE increases mitochondrial membrane potential in murine HFs. Skin biopsies collected from mice treated with AAE or placebo foam were incubated ex-vivo with Mitotracker CMX-ROS. UPPER PANEL: A faint fluorescence emission of the probe (red channel) is detectable in Keratin 14 positive and Keratin 6 HF cells (green channel) in placebo biopsies. LOWER PANEL: Increased fluorescence emission of Mitotracker CMX-ROS in Keratin 14 and Keratin 6 positive HF cells and hair bulges of mice treated with AAE. Asterisks indicate HFs. Arrows indicate bulge regions. Scale bars correspond to 200 μm in (**a**), (**c**) and (**d**), 50 μm in (**b**).

To further prove that the treatment with Annurca Polyphenols was indeed increasing mitochondrial activity in HFs, we probed skin biopsies of mice topically treated with AAE with the mitochondrial probe MitoTracker CMX-ROS. The fluorescence emitted by this dye correlates with the membrane potential of the mitochondrial intermembrane space. The latter, depending on the number of protons transported by the electron transport chain, is a direct measurement of mitochondrial activity. The dye faintly stained HFs of mice treated with the foam containing the placebo (Figure 3, upper panel), confirming, as already reported [46,47] a low mitochondrial activity in telogen HFs [40]. On the contrary, in AAE-treated HFs, the fluorescence emitted by the dye appeared much more intense, confirming an increased mitochondrial activity (Figure 3, lower panel). We could visualize the fluorescence of the dye mostly in bulge cells and in ORS cells (Keratin 14 and Keratin 6 positive HF cells) and only rarely in those located in other regions of the HF.

Overall our metabolomic analysis reveal that glycogenolysis, glutaminolysis, PPP, glutathione and nucleotide synthesis are all diminished in vivo by the treatment with Annurca Polyphenols. On the contrary, mitochondrial respiration, β-oxidation and keratin production are stimulated by the treatment with AAE. In the presence of this alternative source of ATP, HFs probably spare amino acids, avoiding them from being oxidized, and keeping them available for keratin production.

## 4. Discussion

Annurca Apples have shown their potential as nutraceutical in many biological contexts. The plethora of specific and peculiar intracellular pathways activated by this cultivar seems to depend on the high amount of Procyanidin B2 they contain. Annurca Apples polyphenols act as antioxidant, modulate in vivo lipid metabolism and cholesterol levels and act against stress and aging [20,50,51,52]. Despite the different biological system in which AAE resulted to be active, the identification of the molecular mechanism underpinning its activity is far from being complete. Addressing this question is not a simple task. The Polyphenolic fraction of Annurca contains hundreds of different metabolites [26,53]. The activity of most of the polyphenols contained in AAE is drastically reduced when they are isolated from the extract and used as pure molecules [54]. AAE phytocomplex seems to represent the perfect environment where polyphenols can exert their biological function. The phytocomplex probably acts chemically, by protecting apple metabolites from oxidation and degradation. Recent evidences may suggest that the phytocomplex may as well act biologically by activating and deactivating specific cellular pathways, ultimately augmenting the efficacy of the active metabolites [54]. In this scenario, the need to follow hundreds of compounds simultaneously increases the complexity of any study aiming at the identification of the mechanism of action of nutraceuticals. Here, the use of an in vivo system and the metabolite profiles of HFs depicted by high resolution mass spectrometry technique, allowed us to take a snapshot of some of the metabolic pathways activated by Annurca polyphenols in HFs. We are, of course, not sure to have recovered by hair plucking all the different cell lineages present in HFs. We have surely analyzed CD34, Keratin 15, Lgr5 and GLI-1 positive HF cells and thus covered most of the cell lineages present in telogen and early anagen [55] However, their specific contribution to the full metabolic profile we here present is not possible to determine. Clearly, the picture we obtained is not yet complete, but it is useful to explain some of the hair growth promoting effects of Annurca Polyphenols.

**Figure 4:**
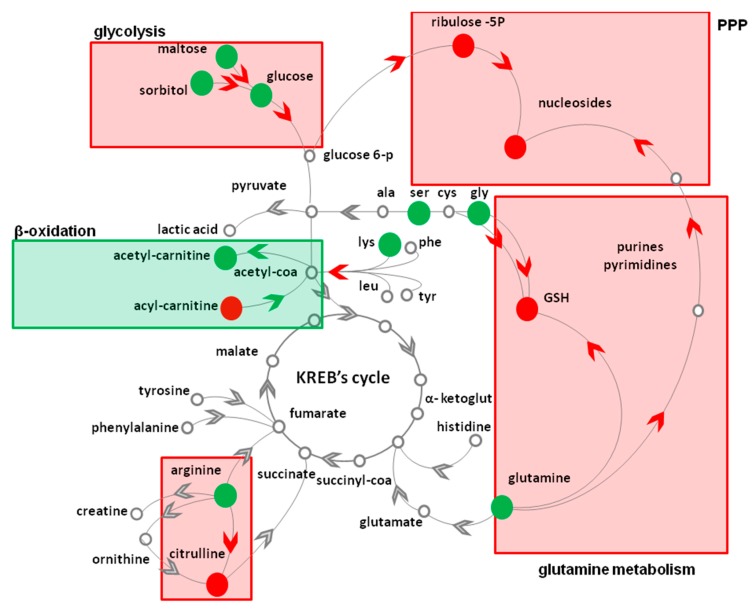
Topical treatment with AAE alters the murine HF metabolome. Schematic cartoon depicting some of the metabolic reactions positively (green box) or negatively (red boxes) affected by AAE in murine HFs. Red and green arrowheads indicate reactions halted or stimulated by AAE, respectively. Red and green dots indicate metabolites whose intracellular concentration resulted decreased or increased by treatment with AAE, respectively.

Our metabolite profiling (Table 1) revealed that the topical treatment for 4 weeks with a cosmetic foam containing AAE significantly changed the levels of at least 18 key intracellular metabolites. The (i) significant elevation of glucose, (ii) glutamine, (iii) glycine, (iv) the increase in the intracellular level of the PPP intermediate ribulose 5P together with (v) the reduction of the intracellular level nucleotides and deoxy-nucleotides all together suggest that AAE causes a reduction in the utilization of glucose and glutamine for PPP, a metabolic pathway that in HFs, supports nucleotide biosynthesis. Interestingly, all the reaction involved seems to share NADPH as cofactor. This electron carrier is involved in many anabolic reactions including synthesis of ribose 5P, of nitrogen-containing bases and of nucleotides. AAE probably affects the overall redox environment of the cell, affecting NADPH production and redox potential. The reduction of the intracellular level of citrulline, the only catabolite of arginine requiring NADPH for its production and the reduced intracellular level of GSH seems to confirm and support our hypothesis that NADPH dependent reaction are halted in AAE-treated HFs (Figure 4).

Metabolic profiling reveals that, upon AAE, HFs engage in mitochondrial β-oxidation as indicated by the reduced levels of one of its substrate palmitoyl-carnitine. Consistently we measured an increase in acetyl-carnitine, indicative of high amounts of Acetyl-CoA in HFs. In Keratin 6 and Keratin 14-positive bulge cells we measured an increased mitochondrial membrane potential confirming the activity of these organelles in AAE stimulated HFs. Mitochondria have been shown to contribute to HF energy metabolism. Genetic disruption of the mitochondrial electron transport chain has proven that mitochondrial function is essential for maintenance of HF. L-carnitine, which promotes mitochondrial β-oxidation, favorably affects hair growth in vitro [48] and in vivo [47]. Mitochondria are also important in follicle morphogenesis, because keratinocytes mitochondrial DNA depletion decreases hair follicle density, increases apoptosis, and reduces proliferation. Moreover, stimulation of mitochondrial function with thyroid hormones prolongs anagen and modifies intrafollicular keratin expression [56,57,58,59,60].

We cannot be sure if the increase in mitochondrial activity induced by the AAE is “a consequence” of the HF progression from telogen to anagen or if it is, on the contrary, “the reason” for the progression. In support of the first hypothesis, a progressive mitochondrial hyper-polarization has been recently shown in early anagen HF cells. This mitochondrial activity seems to increase in differentiating HF cells and to abruptly reduce when differentiation is complete [46,55,61,62]. However, in support of the second hypothesis, polyphenol have been in many contexts shown to directly affect mitochondrial activity and stimulate mitochondrial ATP production. Both epicatechin and Procyanidin B2 possess uncoupling effect on oxidative phosphorylation in cardiac mitochondria [63], suppressing activity of pyruvate and malate oxidation and stimulating fatty acid β-oxidation. Comparing our metabolic profiles with those described for murine HFs in different stages of the hair cycle [55], it seems more likely that the mitochondrial activity itself is responsible for inducing the early anagen aspect of AAE-treated HFs. More experiments will be needed to clarify this aspect.

Whatever the scenario, HFs treated with AAE are using the electron transport chain for producing energy. Hair growth is a highly energy-consuming process [64,65], and the use of mitochondria for ATP production probably helps sparing glutamine, glycine, and other important amino acids for keratin production from being oxidized.

One puzzling point regards the Wnt inhibitory affect has recently been described for AAE in human colon rectal cancer cells [54]. Wnt pathway activity is essential for HF morphogenesis and regeneration [66]. Valproic acid and lithium chloride, all known Wnt inducers, promote hair regeneration in murine models while the Wnt agonist Wnt7a is showing promising results in Phase I human clinical trials [67,68]. Wnt agonists have been shown to be secreted by DP cells to initiate hair stem cells to HF formation, as well as HM cells to initiate anagen [69]. In the process of becoming HM cells hair stem cell engages in aerobic glycolysis, lactate production [55] and PPP to sustain the high rate of proliferation they are subjected to. How does the Wnt inhibitory activity of AAE not interfere with its hair-promoting effect in HFs? A simple explanation could be found considering the many biological differences between colonocites and HFs. On the other hand, Wnt activity has been shown to be only transiently activated during telogen to anagen transition [70,71]. We show that AAE reduces the expression level of *CD34*, a marker of quiescent stem cells, while it does not alter the levels of the Wnt target gene *Lgr5*. These preliminary results could point toward a scenario where Wnt inhibitory activity of AAE contributes to the metabolic reprogramming of HFs by sustaining and accelerating transition of quiescent stem cells into differentiated keratin producing cells [60,61,62].

All we have described must be added to the already proven antioxidant activity of AAE [20]. Oxidative stress has been shown to promote the catagen phase in HFs [72] and pro-oxidant effects are suggested as the major mediators of smoking and ultraviolet light induced exacerbation of PHL and Androgenic Alopecia [73]. AAE anti-oxidant potential is surely contributing to hair growth by protecting HFs from environmental oxidative stress.

## 5. Conclusions

While significant effort has produced a wealth of knowledge on the transcriptional and epigenetic mechanism by which apple polyphenols exert their activity, little is known about the effect they exert on metabolic pathways in vivo. Since there are essentially no published data on the effect of polyphenols on the metabolic states of any cell in the HFs, a metabolomic study was necessary to understand the effect of the extract and suggest a possible explanation for it. Together with their already describe anti-oxidant potential we here show that AAE greatly affect mitochondria of HFs. HFs stimulated by AAE engage in fatty acid β-oxidation to generate ATP, sparing amino-acid from catabolism, ultimately igniting keratin production.

## Figures and Tables

**Table 1:** Fold induction for the indicated metabolites measured upon in vivo treatment with AAE.

Metabolic Pathway	Metabolite	Fold Change ^1^	Metabolic Pathway	Metabolite	Fold Change ^1^
Glycolysis			PPP		
	Glucose *	2.4 ± 0.2		Ribulose 5P *	3.2 ± 0.1
	Lactic acid	1.1 ± 0.2			
Glycogenolysis			Nucleotides	Adenosine *	0.4 ± 0.1
	Maltose	2.2 ± 0.1		Cytidine *	0.6 ± 0.1
	Sorbitol	3.1 ± 0.2		Deoxy-cytidine *	0.5 ± 0.1
Amino acids				Deoxy-inosine *	0.5 ± 0.1
	Glutamine *	2.4 ± 0.2			
	Glycine	6.2 ± 0.2	β-oxidation	Palmitoyl-carnitine	0.8 ± 0.1
	Serine	1.3 ± 0.2		Acetyl-carnitine	1.6 ± 0.2
	Lysine	1.3 ± 0.2			
	GSH *	0.9 ± 0.1			
	Arginine	1.3 ± 0.2			
	Citrulline *	0.7 ± 0.2			

^1^ (*n* = 15. Shown is mean ± SEM); * indicates metabolites requiring NADPH for their anabolism/catabolism.

## Supplementary Materials

The following data are available online at http://www.mdpi.com/2072-6643/10/10/1406/s1, Figure S1: Effect of AAE on HFs measured by qPCR analysis, Figure S2: Partial least squares-discriminant analysis (PLS-DA) of HFs metabolites determined by FT-ICR–MS, Table S1: Identification of metabolites in HFs determined by DI- FT-ICR-MS.
