# Supplementary material for: Annurca Apple Polyphenols Ignite Keratin Production in Hair Follicles by Inhibiting the Pentose Phosphate Pathway and Amino Acid Oxidation
Source: Nutrients. 2018 Oct 2;10(10):1406. doi: 10.3390/nu10101406 (PMC6213762; doi:10.3390/nu10101406)
Supplement: Supplementary file 1 [file nutrients-10-01406-s001.pdf]

Supplementary material for the manuscript

Annurca Apple Polyphenols ignite Keratin production in Hair Follicles by inhibiting Pentose Phosphate Pathway and Amino Acid Oxidation.

Nadia Badolati [1†], Eduardo Sommella [2†], Gennaro Riccio [1], Emanuela Salviati [2, 3], Dimitri Heintz [4], Sara Bottone [1], Emery Di Cicco [5], Monica Dentice [5], Giancarlo Tenore [1], Pietro Campiglia [2], Mariano Stornaiuolo [1, *] and Ettore Novellino [1,*]

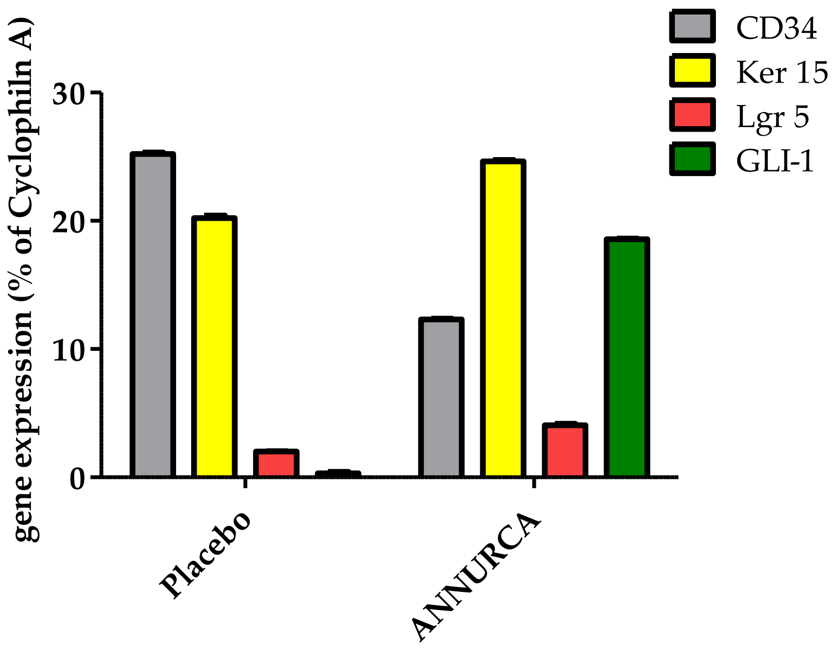

Figure S1. Effect of AAE on HFs measured by qPCR. Effect exerted by AAE on the expression of HF stem cell markers *CD34*, *Keratin 15* (K15), *Lgr5* and *GLI-1* in HFs treated with Placebo or with ANNURCA analyzed by qPCR. Values are expressed as % of mRNA compared to those of internal standard *cyclophilin A*. Experimental procedures and statistics as described in the Methods Section and in Figure 2.

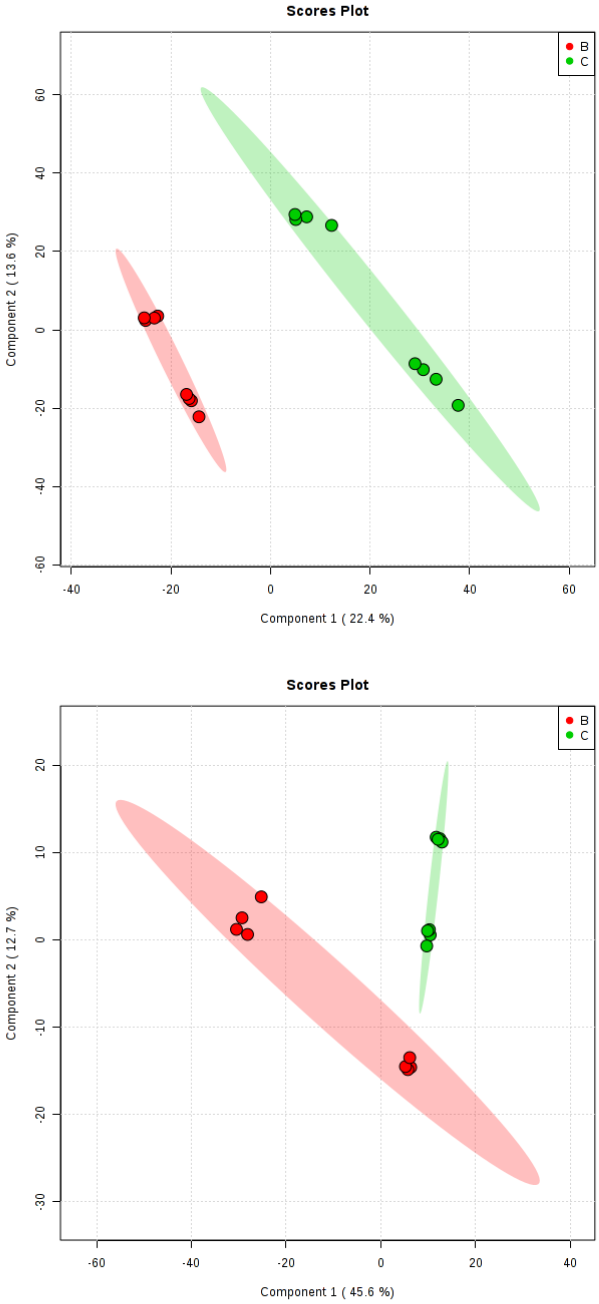

Figure S2 a (top), b (bottom): Partial least squares-discriminant analysis (PLS-DA) of HFs metabolites determined by FT-ICR–MS, the two dimensional score plots show clustering and separation between AAE treated mice (green symbols) and Placebo (red symbols). Ellipses represent 95% confidence intervals.

Metabolite	m/z	Ionization	Error (ppm)
Glucose	203.05265	$[M+Na]^+$	0.404
Lactic acid	113.02091	$[M+Na]^+$	-0.176
Maltose	365.10543	$[M+Na]^+$	-0.045
Sorbitol	205.06834	$[M+Na]^+$	0.395
Glutamine	169.05836	$[M+Na]^+$	0.001
Glycine	113.99511	$[M+K]^+$	-0.682
Arginine	197.10090	$[M+Na]^+$	0.029
Serine	144.00584	$[M+K]^+$	0.607
Lysine	147.11288	$[M+H]^+$	0.515
GSH	306.07675	$[M-H]^-$	0.719
Citrulline	198.08495	$[M+Na]^+$	0.072
Ribulose-5-P	233.04228	$[M+H]^+$	0.856
Adenosine	290.08596	$[M+Na]^+$	-0.063
Cytidine	266.07476	$[M+Na]^+$	0.100
Deoxy-Cytidine	250.07984	$[M+Na]^+$	0.044
Deoxy-Inosine	275.07507	$[M+Na]^+$	-0.091
Palmitoyl-carnitine	422.32404	$[M+Na]^+$	0.148
Acetyl-carnitine	226.10501	$[M+Na]^+$	0.211

Table S1: Identification of metabolites in HFs determined by DI- FT-ICR-MS
